# Adolescent Pregnancy in Southeastern Romania: A Ten-Year Retrospective Cohort from a Regional Referral Center

**DOI:** 10.3390/medicina61122162

**Published:** 2025-12-04

**Authors:** Dragoș Brezeanu, Ana-Maria Brezeanu, Simona Stase, Vlad-Iustin Tica

**Affiliations:** 1 6th Department, Faculty of Medcine, Ovidius University of Constanta, 900470 Constanta, Romania; brezeanudragos@gmail.com (D.B.); stase.simona.10d@gmail.com (S.S.); vtica@eeirh.org (V.-I.T.); 2 Obstetrics Department, County Clinical Emergency Hospital ”Sf. Ap. Andrei”, 900591 Constanta, Romania

**Keywords:** teenage pregnancy, cesarean section, adolescent health, reproductive outcomes, socioeconomic status, public health

## Abstract

*Background and Objectives*: Adolescent pregnancy remains a major global public-health concern, particularly in low- and middle-income countries. Romania consistently reports the highest teenage birth rate in the European Union, with pronounced regional disparities. This study aimed to assess the incidence, sociodemographic predictors, and obstetric outcomes of adolescent pregnancies over a ten-year period in southeastern Romania. *Materials and Methods*: A retrospective, population-based study was conducted at the Clinical County Hospital “Saint Andrew the Apostle”, Constanța, from 1 January 2014 to 31 December 2023. All deliveries involving mothers aged ≤19 years were identified from institutional databases. Demographic variables (age, residence, education) and obstetric outcomes (mode of delivery, gestational age, neonatal parameters) were analyzed. Statistical tests included Pearson’s χ^2^, *t*-tests or Mann–Whitney U tests, and multivariate logistic regression to identify independent predictors of cesarean delivery and preterm birth. *Results:* Among 13,416 hospital deliveries, 1640 (12.2%) involved adolescent mothers (mean age 16.3 ± 1.4 years). Most originated from rural areas (64.6%) and had only primary education (42.8%). Cesarean section was performed in 58.3% of cases—significantly higher among rural (61.2%) and low-education (62.4%) groups (*p* < 0.05). The leading indications were cervical dystocia (19.2%) and cephalopelvic disproportion (16.9%). Preterm birth occurred in 30.5% and low birth weight in 27.1% of neonates. Multivariate analysis identified primiparity (OR 2.10; 95% CI 1.45–3.05; *p* < 0.001) and low education (OR 1.56; 95% CI 1.09–2.21; *p* = 0.015) as independent predictors of cesarean delivery, while rural residence and low education predicted prematurity (OR 1.84; 95% CI 1.12–3.02; *p* = 0.016). *Conclusions*: Adolescent pregnancy in southeastern Romania remains a persistent public-health challenge concentrated among rural and low-education populations. These patterns are consistent with previously described vulnerabilities in adolescent populations, including developmental and healthcare-access challenges, although such factors were not directly measured in this study. Community-based prevention, comprehensive sexual education, and adolescent-friendly obstetric pathways are urgently needed to reduce the burden of teenage pregnancy in Eastern Europe. These associations should be interpreted with caution, as the retrospective design precludes causal inference.

## 1. Introduction

Adolescent pregnancy remains a persistent and complex challenge for global public health, intersecting developmental vulnerabilities, socioeconomic disadvantage and broader health-system challenges described in the previous literature [1,2]. According to the World Health Organization (WHO), the global adolescent birth rate (ages 15–19) has declined from 64.5 births per 1000 women in 2000 to 41.3 births per 1000 in 2023; however, this decline has been uneven across regions, and many adolescents remain at elevated risk of adverse outcomes [3].

Adolescent mothers are disproportionately represented among those with limited education, low socioeconomic status, rural residence and inadequate prenatal care [4,5].

From a biological and obstetric viewpoint, previous studies suggest that incomplete physical maturation may contribute to higher rates of preterm birth, low birth weight, and intrapartum complications among adolescents, although such mechanisms were not directly evaluated in the present study, which may increase the incidence of preterm birth, low birth weight, fetal growth restriction and obstetric complications such as cephalopelvic disproportion, dystocia and hypertensive disorders [6,7].

A systematic review revealed that adolescent pregnancies were strongly associated with less favorable neonatal and maternal outcomes, including prematurity, small for gestational age neonates, and increased cesarean delivery rates [8].

In addition to clinical outcomes, adolescent pregnancy is embedded in sociostructural processes: early childbearing may interrupt education, limit economic opportunities, perpetuate cycles of poverty, and reflect gender inequalities and limited reproductive autonomy [9].

In the European Union context, particularly in Eastern Europe, the prevalence of adolescent births remains higher than that in many Western European countries, with Romania having among the highest national rates in Europe [10,11].

In Romania, large-scale reports highlight that adolescent mothers (under age 20) represent approximately 10% of births and that their social disadvantage—rural residence, low parental education, early school leaving—is strongly implicated in their risk profile [12].

Importantly, although the global literature on adolescent pregnancy is extensive, there remains a significant gap in region-specific, population-based studies that link sociodemographic predictors (such as rural vs. urban residence, educational level, and maternal age at first birth) with detailed obstetric and perinatal outcomes in Eastern Europe. Many studies rely on descriptive data; fewer provide multiyear cohorts, adjusted analyses, and an explicit context of health-system factors [13]. This is particularly true for southeastern Romania, where regional disparities, rural–urban differences, and healthcare access issues may modify the classic risk profile of adolescent pregnancy [14,15].

The present study addresses this gap by analyzing a ten-year cohort (2014–2023) of adolescent pregnancies (≤19 years) at a major referral hospital in southeastern Romania (Constanța County). Specifically, the study quantifies the incidence of adolescent pregnancy, describes the sociodemographic profile (maternal age, residence, educational attainment), and examines obstetric outcomes, including mode of delivery (cesarean vs. vaginal), Section 3.4, and key perinatal outcomes (prematurity, low birth weight, neonatal complications). In so doing, this study aims to provide evidence relevant for clinical practice and public health policy tailored to the Romanian context while contributing to the international literature on adolescent pregnancy by offering region-specific data and analyses.

Despite Romania having one of the highest adolescent birth rates in the European Union, published evidence from Southeastern Romania remains limited to small cross-sectional reports with insufficient follow-up duration and minimal analytical depth. Existing studies rarely differentiate regional patterns, and none provide long-term, multivariate analyses linking sociodemographic characteristics with detailed obstetric outcomes among adolescents [16]. This lack of regionally stratified evidence restricts understanding of population-specific vulnerabilities and limits the development of targeted public health strategies. To address this gap, the present study analyzes a ten-year hospital-based cohort of adolescent pregnancies from a major tertiary referral center in Southeastern Romania, using multivariate methods to examine associations between demographic factors and key obstetric outcomes. This approach provides one of the most comprehensive regional datasets to date and contributes novel epidemiological insight into adolescent pregnancy in this understudied area.

We hypothesize that in this cohort, adolescent mothers from rural areas and with lower educational attainment will exhibit higher rates of cesarean delivery, prematurity and neonatal complications than adolescents from urban areas or with higher education. The objectives of this study are as follows:

1. To determine the incidence of adolescent pregnancy (≤19 years) in the 2014–2023 period at the study hospital.
2. To identify the sociodemographic distribution of adolescent mothers (residence, educational level, maternal age) in the cohort.
3. To assess the associations between sociodemographic predictors and obstetric/perinatal outcomes (e.g., mode of delivery, prematurity, neonatal complications).
4. To discuss the implications for the clinical management of adolescent pregnancies and for targeted interventions in public health and reproductive services in Romania.

## 2. Materials and Methods

### 2.1. Study Design and Setting

This retrospective, population-based observational study was conducted at the Clinical County Hospital “Saint Andrew the Apostle” in Constanța, Romania. The institution is a tertiary referral center and the main obstetric facility for the southeastern region of the country, providing maternity care for both urban and extensive rural populations.

The analysis covered a continuous ten-year interval, from 1 January 2014 to 31 December 2023, during which time all obstetric and gynecological admissions were reviewed. The study was designed and reported in accordance with the Strengthening the Reporting of Observational Studies in Epidemiology (STROBE) guidelines.

### 2.2. Patient Selection and Data Validation

The process of patient identification and selection was conducted in several structured stages to ensure data reliability and compliance with the predefined eligibility criteria.

**Figure 1:**
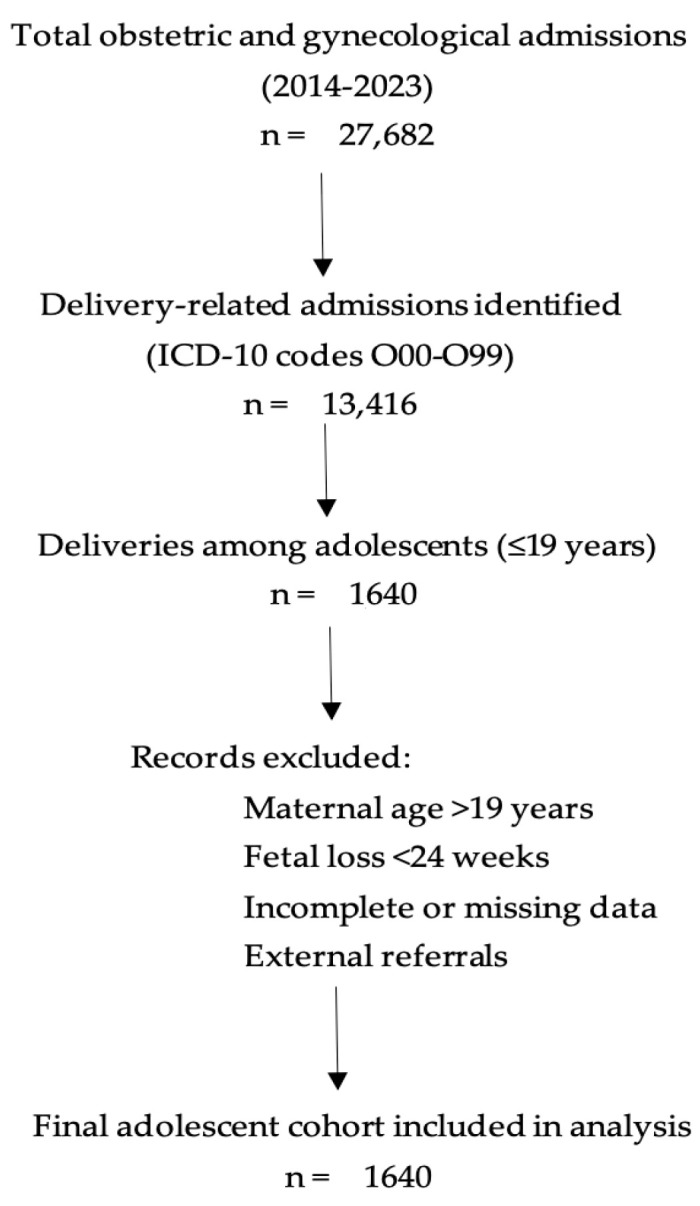
STROBE flowchart.

First, the hospital’s electronic medical archive was queried for all obstetric admissions registered between 1 January 2014 and 31 December 2023. Using a combination of International Classification of Diseases (ICD-10) codes corresponding to pregnancy, childbirth, and the puerperium (O00–O99), a preliminary dataset of 13416 delivery-related admissions was generated (Figure 1).

STROBE flowchart- illustrating the identification, screening, exclusion, and inclusion of adolescent pregnancies (≤19 years) at the County Clinical Emergency Hospital “Saint Andrew the Apostle”, Constanța, during the 2014–2023 period. From a total of 27,682 obstetric and gynecologic admissions, 13,416 childbirth-related cases were initially identified. After filtering by maternal age (≤19 years), 1640 cases met the preliminary inclusion criteria.

In the second stage, records were filtered by maternal age to isolate all deliveries involving patients aged 12–19 years at the time of birth. Age verification was based on each patient’s national identification number, which contains encoded date-of-birth data and was cross-checked against the admission registry. This procedure yielded a cohort of 1640 adolescent pregnancies, representing 12.2% of all deliveries during the study period.

Each medical record was subsequently individually reviewed by two independent investigators (A.M.B. and S.S.) to confirm eligibility according to the inclusion and exclusion criteria. Discrepancies were resolved through joint re-examination and, when necessary, consultation with a third senior reviewer (V.-I.T.). This dual-review process minimized classification bias and ensured uniform application of definitions across all cases.

Data completeness was assessed before analysis. A total of 68 records (4.14%) were excluded due to incomplete documentation, defined as missing core obstetric variables required for analysis. The most frequently missing variables were gestational age at delivery and neonatal birthweight, which prevented reliable classification of key perinatal outcomes. No imputation procedures were applied, and only complete cases were included in the analytical dataset.

### 2.3. Inclusion Criteria and Exclusion Criteria

#### 2.3.1. Inclusion Criteria

Eligible participants were selected according to the following criteria:

Age at delivery: All female patients aged between 12 and 19 years at the time of delivery were considered eligible. Age verification was based on the birth certificate or official identification document recorded in the hospital database.

Type of pregnancy: Both singleton and multiple gestations were included, regardless of parity or gravidity, provided that the pregnancy resulted in a hospital delivery or a pregnancy-related admission.

Gestational age: No lower limit was imposed for gestational age at admission, allowing for the inclusion of preterm and post-term deliveries, as well as pregnancies complicated by early preterm labor.

Place of care: Only patients who delivered or were admitted for pregnancy-related management at the Department of Obstetrics and Gynecology II, Clinical County Hospital “Saint Andrew the Apostle”, Constanța, during the study period (1 January 2014–31 December 2023) were eligible.

Data completeness: Eligibility required the availability of complete medical and demographic information, including admission records, delivery reports, and neonatal outcomes, as documented in the hospital’s electronic medical system.

#### 2.3.2. Exclusion Criteria

Patients meeting any of the following conditions were excluded from analysis:

Maternal age above 19 years: Deliveries among women aged over 19 years were excluded to maintain a strict focus on the adolescent population as defined by the World Health Organization.

Early pregnancy losses, such as miscarriages, elective terminations, or intrauterine fetal demises occurring before 24 completed weeks of gestation, were not included, as these do not meet the operational definition of delivery and often lack comparable obstetric data. Non-standard analgesia methods or previous obstetrical abnormalities (in case of multipara)—as we have previously reported—were also reasons for exclusion [17,18,19,20].

Incomplete documentation: Records with missing key variables—such as gestational age at birth, mode of delivery, or neonatal outcome—were excluded to ensure data integrity and statistical validity.

External referrals: Patients transferred from other medical facilities whose records lacked complete obstetric documentation (e.g., prenatal history, labor details, or operative notes) were excluded to avoid duplication and inconsistencies across institutional data systems.

### 2.4. Operational Definitions

For the purposes of this analysis, teenage pregnancy was defined as any pregnancy occurring in a female aged ≤19 years at the time of delivery. Preterm birth referred to delivery before 37 completed weeks of gestation.

Low educational attainment was defined as incomplete primary or lower secondary education (≤8 years of schooling). Educational attainment was extracted from the hospital admission file, where it is routinely recorded based on the patient’s official declaration and verified, when available, against accompanying identity documents or school certificates. In cases where documentary proof was not provided, the education level was registered as self-reported, following standard hospital protocol.

The cesarean section rate (CSR) was calculated as the proportion of cesarean deliveries among all deliveries within the adolescent cohort.

### 2.5. Statistical Analysis

Statistical analysis followed a structured, stepwise approach, in accordance with recommended reporting principles for transparency and reproducibility in medical research [21]. First, descriptive statistics were used to summarize demographic and clinical characteristics, expressed as means with standard deviations for continuous variables and frequencies with percentages for categorical variables. Second, the annual incidence of adolescent pregnancy was calculated for each year of the study period, and trends were evaluated using the chi-square test for trend.

Third, bivariate analyses were conducted to explore crude associations between each variable and mode of delivery. Categorical variables were compared using the chi-square test or Fisher’s exact test, and continuous variables using the independent-samples *t*-test. Variables with a *p*-value < 0.10 in the bivariate analysis were retained for multivariate testing.

Fourth, a multivariate logistic regression model was constructed to estimate the adjusted odds of cesarean delivery. All eligible variables were entered simultaneously using the enter method. To assess potential multicollinearity, we calculated variance inflation factors (VIFs), considering values ≥ 5 as indicative of problematic collinearity.

Fifth, overall model performance was evaluated using the Hosmer–Lemeshow goodness-of-fit test and the Nagelkerke R^2^ statistic. Adjusted odds ratios (aORs) with 95% confidence intervals (95% CIs) were reported.

Statistical significance was set at α = 0.05. Analyses were performed using SPSS version 26.0 (IBM Corp., Armonk, NY, USA).

### 2.6. Ethical Considerations

The study protocol was approved by the Ethics Committee of the Clinical County Hospital “Saint Andrew the Apostle” Constanța (approval no. 45/2024).

The investigation was conducted in full compliance with the Declaration of Helsinki and national ethical regulations governing retrospective research.

Because the study involved secondary analysis of anonymized data, the requirement for individual informed consent was waived. All patients admitted to the hospital had previously signed general consent forms authorizing the use of deidentified clinical data for research purposes.

## 3. Results

### 3.1. Overall Incidence and Demographic Profile

During the ten-year study period (2014–2023), 27,682 admissions were recorded in the Department of Obstetrics and Gynecology at the Clinical County Hospital “Saint Andrew the Apostle”, Constanța. Among these, 13,416 admissions (48.5%) were related to childbirth, whereas 1640 (12.2%) involved adolescent pregnancies (≤19 years of age). The mean maternal age was 16.3 ± 1.4 years (range 12–19) (Table 1). Of the initial dataset, 68 cases (4.14%) were excluded due to missing core obstetric information—primarily birthweight and gestational age—resulting in a final analytic sample of 1640 adolescent pregnancies.

Sociodemographic profile of adolescent mothers (*n* = 1640), showing a predominantly rural, low-education, early adolescent cohort with a narrow age distribution.

A clear sociodemographic imbalance was observed. Nearly two-thirds of the adolescents originated from rural communities (64.6%), and the remainder originated from urban settings (35.4%). Educational attainment was markedly low: 42.8% had completed only the primary cycle (1–4 years of schooling), 23.4% had reached lower secondary education, and only 33.8% were enrolled in or had completed the upper secondary level (Table 1). This distribution reflects the social vulnerabilities that characterize adolescent pregnancy in the region.

### 3.2. Temporal Distribution

**Figure 2:**
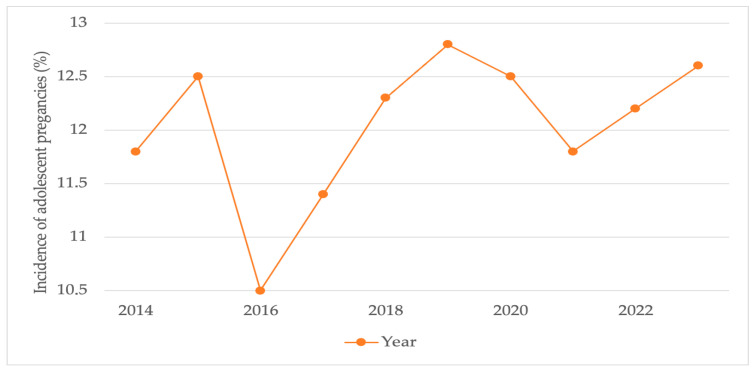
Annual incidence of adolescent pregnancy (2014–2023).

The annual incidence of teenage pregnancy remained relatively stable over the 10-year observation period (Table 2 and Figure 2). The highest rate was recorded in 2019 (12.8%), whereas the lowest rate occurred in 2016 (10.5%). The year-to-year variation did not reach statistical significance (χ^2^ = 3.1, *p* = 0.96), indicating that adolescent pregnancy represents a persistent rather than episodic public health issue within this population.

Data were extracted from institutional birth records (2014–2023). The incidence was calculated as the proportion of teenage deliveries (<19 years) relative to total annual births. The χ^2^ test for trend revealed no significant variation across years (χ^2^ = 3.1, *p* = 0.96).

Annual incidence of adolescent pregnancies (≤19 years) during the 2014–2023 period at the County Clinical Emergency Hospital “Saint Andrew the Apostle”, Constanța. Year-to-year variation was not statistically significant (χ^2^ = 3.1, *p* = 0.96; 95% CI overlap across years).

### 3.3. Mode of Delivery

Among the 1640 deliveries analyzed, 956 (58.3%) were completed by cesarean section, whereas 684 (41.7%) were vaginal births. The cesarean section rate among adolescents substantially exceeded the national average for all age groups (~44%), highlighting a clinically relevant obstetric burden.

Cesarean delivery was significantly more common among rural residents (61.2%) than among their urban counterparts (52.4%; *p* = 0.02). A similar trend was observed across educational strata: adolescents with primary education presented a higher cesarean rate (62.4%) than did those with secondary or upper secondary schooling (49.1%; *p* = 0.01) (Table 3).

Cesarean delivery predominated in the cohort, accounting for 58% of births, with higher rates among adolescents from rural areas and those with lower educational attainment.

When educational attainment was examined across three distinct categories, a graded pattern became evident. Adolescents with primary education had the highest cesarean rate (62.4%), followed by those with lower secondary education (57.7%), while the lowest rate was observed among adolescents with upper secondary education (54.4%). The difference across groups was statistically significant (*p* = 0.01), suggesting an inverse association between years of schooling and mode of delivery. These descriptive findings provide additional granularity beyond the binary categorization used in the regression model.

### 3.4. Indications for Cesarean Section

The predominant indications for cesarean delivery were cervical dystocia (19.2%), cephalopelvic disproportion (16.9%), and breech presentation (7.7%), a pattern consistent with prior literature suggesting that pelvic developmental factors may contribute to operative delivery among younger adolescents, although such aspects were not directly assessed in this study. (Table 4).

A further 13.6% of the cesarean sections were performed preterm (<37 weeks), primarily due to fetal distress (4.8%) or premature rupture of membranes (PROM) (3.6%).

A previous uterine scar was documented in 25.3% of cases, a proportion consistent with the regional pattern of repeated cesarean deliveries in young multiparas.

The leading indications for cesarean delivery were a previous uterine scar (25.3%), cervical dystocia (19.2%), and cephalopelvic disproportion (16.9%), together representing more than 60% of all cases.

### 3.5. Perinatal Outcomes

The incidence of preterm birth (<37 weeks) was 30.5% (*n* = 500), which was more than double the national mean. Low birth weight (<2500 g) occurred in 27.1% of the newborns, and the mean birth weight was 2740 ± 510 g.

An Apgar score < 7 at 5 min was observed in 8.9% of the neonates, predominantly among those with preterm deliveries (*p* < 0.001). Neonatal intensive care unit (NICU) admission was required for 11.6% of the patients, most frequently because of respiratory distress or complications of prematurity (Table 5).

Multivariate analysis demonstrated that both rural residence and low educational attainment independently predicted preterm delivery (OR = 1.84; 95% CI: 1.12–3.02; *p* = 0.016). These findings underscore the cumulative impact of socioeconomic disadvantage on neonatal vulnerability.

Preterm birth and low birth weight were the most frequent adverse outcomes, with strong associations to rural residence, low education, and prematurity.

### 3.6. Variables Associated with Higher Odds of Cesarean Delivery

Binary logistic regression identified primiparity and low educational attainment as the strongest independent predictors of cesarean section (Table 6). After adjustment for maternal age and residence, primiparous adolescents had twice the odds of cesarean delivery compared with their multiparous peers (OR = 2.10; 95% CI: 1.45–3.05; *p* < 0.001). Similarly, adolescents with only primary education were 56% more likely to have a cesarean section (OR = 1.56; 95% CI: 1.09–2.21; *p* = 0.015) (Table 6).

The logistic regression model demonstrated acceptable fit, with a Hosmer–Lemeshow test *p*-value of 0.41 and a Nagelkerke R^2^ of 0.19, indicating moderate explanatory power. Variance inflation factors were below 2 for all variables, suggesting no problematic multicollinearity.

Neither gestational age at birth nor rural residence remained statistically significant after adjustment.

Primiparity and low education were the only independent variables of cesarean delivery.

### 3.7. Synthesis of Findings

Across the ten-year period, adolescent pregnancies represented a stable yet disproportionate share of all births in Constanța County. The cohort was characterized by pronounced social disadvantage, reflected in rural predominance and limited educational attainment.

The elevated rates of cesarean delivery and preterm birth observed in this cohort align with patterns previously reported among adolescent mothers, who often face developmental, behavioral, and healthcare-access challenges. These contextual interpretations are supported by external literature; however, our study did not directly measure biological maturation, provider-level practices, or prenatal care utilization.

## 4. Discussion

This ten-year population-based investigation provides updated evidence on the magnitude and determinants of adolescent pregnancy in southeastern Romania. The incidence of 12.2% remains far above the European mean and reflects a population shaped by strong rural predominance (65%) and low educational attainment (43% with only primary schooling). These social determinants were closely linked to unfavorable obstetric outcomes, including a 58% cesarean section rate and a 30% prevalence of preterm birth. Such findings position adolescent pregnancy not only as an obstetric concern, but also as a marker of structural inequity and insufficient investment in reproductive health.

Low educational attainment and primiparity were associated with increased odds of cesarean delivery, consistent with international reports connecting socioeconomic disadvantage, reduced health literacy, and delayed prenatal engagement with operative delivery [19]. Although rural residence did not remain significant after adjustment, its overlap with education suggests that these variables capture interconnected socioeconomic dimensions rather than independent pathways. When educational attainment was subdivided into three categories, adolescents with only primary schooling showed the highest cesarean rates, indicating a potential educational gradient that merits further prospective investigation. The small proportion of multiple pregnancies (≈5%), a group inherently at higher risk for prematurity and low birth weight, may have slightly inflated absolute outcome rates, although stratified analyses were not feasible due to sample-size constraints.

Globally, adolescent fertility has declined from 64.5 births per 1000 girls aged 15–19 years in 2000 to approximately 41 per 1000 in 2023 [3,18], yet reductions have been slower in Central and Eastern Europe. Eurostat data continue to position Romania among regional outliers, with national rates exceeding 20 per 1000—three times the European average [22]. Our temporal analysis revealed no significant downward trend from 2014 to 2023, suggesting that existing preventive measures have not yet translated into measurable population-level effects. The small peak in 2020 is consistent with international reports linking pandemic-related school closures and reduced access to reproductive health services with temporary rises in adolescent conception [23,24].

The 58% cesarean section rate substantially exceeds the WHO recommendation of 15–20% and surpasses the Romanian national average of 44% for adolescents. Similar high rates have been documented in low-resource settings (42–55%), although values above 55% remain uncommon [25,26]. Several factors may contribute, including anatomical immaturity predisposing young nulliparas to dystocia or cephalopelvic disproportion—indications that accounted for over one-third of cesareans in our cohort. Structural aspects of the healthcare system may amplify these risks: defensive medical practice, inadequate midwifery support, and limited intrapartum monitoring frequently encourage early recourse to surgery [27,28]. The predominance of cesarean births among rural adolescents suggests additional influences, such as delayed labor presentation and insufficient antenatal risk stratification [29]. International comparisons reinforce the context-dependent nature of operative delivery: Matei et al. reported a 52% cesarean rate among Romanian adolescents, whereas other studies documented 35% in the United States [29,30]. Notably, nearly one-quarter of adolescents in our study had a pre-existing uterine scar, an atypical and concerning feature at this age, further inflating both immediate surgical risk and future reproductive morbidity.

Prematurity and neonatal outcomes mirrored those observed in other high-vulnerability populations. The 30% preterm birth rate, twice the general Romanian rate (≈12–14%) and above the European average (≈10%), aligns with established literature linking adolescent pregnancy with uteroplacental immaturity, anemia, infection susceptibility, and nutritional stress [31,32]. The association between low educational attainment and preterm delivery (OR = 1.84; *p* = 0.016) highlights the behavioral and contextual mechanisms through which disadvantaged adolescents may underutilize prenatal care or underrecognize symptoms. The proportions of low-birth-weight infants (27%) and NICU admissions (11%) were consistent with findings from Sukhopon et al. and Ursache et al. [24,33].

Education emerged as the most influential sociodemographic determinant across outcomes. Adolescents with fewer than eight years of schooling were more likely to experience both cesarean delivery and preterm birth, reflecting well-documented pathways connecting limited reproductive health knowledge, barriers to contraception, and fragmented prenatal engagement [34,35]. These disparities are further shaped by rural residence, where long distances to maternity units, restricted access to contraception, and cultural norms valorizing early motherhood remain influential [36,37,38].

Clinicians caring for adolescent mothers must navigate the intersection of biological immaturity and psychosocial fragility [39,40]. Enhanced antenatal surveillance, early identification of dystocia and structured intrapartum support, including midwifery involvement and continuous monitoring, represent practical strategies to reduce unnecessary cesareans. Postpartum care should incorporate psychological assessment, lactation counseling, and social-work support, given the elevated risk of depression, delayed bonding, and social isolation among young mothers [23,39]. Establishing adolescent-friendly obstetric pathways with dedicated consultation times and follow-up schedules could substantially improve continuity of care.

From a public health perspective, adolescent pregnancy remains a sensitive indicator of social and educational inequity. Sexual education programs in Romania remain fragmented and optional, with UNICEF reporting that fewer than one in five adolescents have received formal instruction on contraception or reproductive consent [10,11]. Evidence from Western Europe and Scandinavia shows that comprehensive sexual education paired with confidential reproductive health services can reduce teenage birth rates by 40–50% within a decade [3,40]. Reintegration programs supporting school return, childcare, and vocational training have demonstrated effectiveness in preventing recurrent pregnancies and improving long-term socioeconomic trajectories [41,42].

Taken together, these findings portray adolescent pregnancy in southeastern Romania as a manifestation of structural vulnerability rather than isolated behavioral factors. Coordinated, multi-level interventions—spanning education, healthcare system strengthening, and social reintegration—are necessary to disrupt the cycle of early motherhood. Future research should transition from prevalence descriptions to intervention-focused designs, including cluster-randomized trials of school-based programs, telehealth reproductive counseling for rural adolescents, and longitudinal cohorts capturing psychosocial trajectories.

Although associations between education, rural residence, and obstetric outcomes were statistically significant, they remain correlational and should be interpreted as hypothesis-generating rather than causal.

The findings of this study underscore the need for coordinated clinical, educational, and policy-level interventions to address adolescent pregnancy more effectively. From a clinical standpoint, obstetricians and midwives should recognize adolescent pregnancies as a distinct risk category requiring early antenatal engagement—with emphasis on nutritional optimization, infection screening, and psychosocial support—as well as continuous labor monitoring and judicious use of cesarean delivery to minimize morbidity and preserve future fertility [19,43,44]. Given the heightened psychological vulnerability of young mothers, integrated postpartum services, including mental health assessment, lactation counseling, and contraceptive guidance, should become standard components of care [19,39,40]. At the community level, comprehensive sexual education combined with confidential reproductive health services remains central to prevention, with evidence showing reductions in teenage conception rates of up to 50% when such programs are fully implemented [3,41,45]. In Romania, expanding these initiatives, particularly in rural schools, through collaboration between educators and family doctors, supported by outreach programs and mobile clinics, is essential [42,46]. At the system level, policymakers should interpret the persistent rates of adolescent pregnancy as indicators of structural gaps rather than isolated behavioral patterns, prioritizing the development of youth-friendly primary care services, educational and socioeconomic reintegration programs for adolescent mothers, national registries for monitoring trends, and enhanced training for healthcare providers in adolescent-centered, stigma-free communication [47,48]. Through such integrated, multi-level measures, adolescent pregnancy can shift from a marker of social inequity to a strategic opportunity for strengthening maternal and child health infrastructure.

### Strengths and Limitations

The principal strength of the present study lies in its large, ten-year, population-level cohort, offering a robust estimate of incidence and outcomes within a defined catchment area. Unlike previous small cross-sectional studies, our dataset integrates both obstetric and sociodemographic variables, enabling multivariate modeling of risk factors.

While the present analysis offers valuable epidemiologic and clinical insights, several limitations must be acknowledged.

First, its retrospective design limits the control over confounding variables and prevents the assessment of temporal causality. The study relied on hospital-based data and therefore may not fully capture pregnancies ending outside the formal healthcare system, such as unregistered births or abortions. Although the proportion of excluded records was low, missing data—particularly for birthweight and gestational age—may have introduced minor selection bias. Because only complete cases were analyzed and no imputation was performed, outcome estimates should be interpreted with this limitation in mind.

Several key variables—such as family income, smoking and alcohol use, nutritional status, contraceptive access, distance to healthcare services, and psychosocial stressors—were not available in the hospital registry and could not be analyzed. Their absence limits the ability to fully contextualize adolescent pregnancy within its socioeconomic and behavioral determinants. Future prospective and multicenter studies incorporating detailed socioeconomic, behavioral, and psychosocial metrics are needed to provide a more complete epidemiological profile.

Third, postnatal follow-up was confined to the immediate hospital stay; long-term outcomes regarding neonatal development, maternal mental health, and social reintegration were not available.

The inclusion of multiple gestations may have led to a slight overestimation of preterm birth and low birth weight rates, as these pregnancies carry inherently higher baseline risks. This limitation should be considered when interpreting the perinatal outcome profile.

Although the sample size allowed for multivariate modeling, no formal power analysis was performed; hence, the study may be underpowered for detecting moderate associations or for controlling type II error. Furthermore, multiple hypothesis testing without correction may have increased the risk of type I statistical error.

A sensitivity analysis stratified by parity was not performed because the number of multiparous adolescents was insufficient to ensure stable or interpretable stratified models. Parity was, however, included as an independent variable in the regression analysis.

As a retrospective, hospital-based observational study, the present analysis can demonstrate statistical associations but not causal relationships between socioeconomic factors and obstetric outcomes. The term “predictor” is therefore used in a statistical sense to indicate variables associated with increased odds of a given outcome, without implying direct causality. Interpretations suggesting mechanistic or sociological impact should be viewed as hypothesis-generating and require confirmation in prospective or interventional designs. Given the retrospective design, all findings represent statistical associations rather than causal effects, and the terminology was revised accordingly to avoid misinterpretation.

In cases of cesarean section, we were unable to stratify patients according to the Robson Classification System to allow a good comparison with other European data, as we do not use this system. This, however, did not alter the result’s validity [49].

Although multicollinearity diagnostics indicated no statistical collinearity between low education and rural residence, these variables conceptually reflect overlapping dimensions of socioeconomic disadvantage. As such, residual confounding is likely, and the observed associations should be interpreted within this broader socioeconomic context.

As this analysis was based on data from a single tertiary hospital, the results reflect regional patterns in Southeastern Romania and cannot be generalized to national adolescent pregnancy trends. Although this hospital covers a large proportion of the regional population, multicenter studies across Romania would strengthen its external validity and allow interregional comparisons. Future multicenter studies incorporating diverse geographic and socioeconomic contexts across Romania are required to ensure broader representativeness.

A comparative analysis between adolescents and adult mothers could not be performed because the database included only adolescent pregnancies. Identifying factors associated with becoming pregnant during adolescence would require a separate study design with an adult comparison cohort and broader socioeconomic and behavioral data. Future research should incorporate matched adult controls to explore determinants of adolescent pregnancy more comprehensively.

Interpretations relating to developmental or systemic factors should be viewed as contextual considerations based on prior research, rather than as variables directly assessed in our dataset.

## 5. Conclusions

This ten-year cohort reveals stable adolescent pregnancy rates, pronounced sociodemographic disparities, and elevated obstetric risks in Southeastern Romania. Educational disadvantage and primiparity were associated with higher cesarean rates, while preterm birth exceeded expected European levels. These findings refine the regional epidemiological picture and underscore the need for broader, multicenter analyses incorporating detailed socioeconomic and healthcare-access data.

## Figures and Tables

**Table 1:** Sociodemographic characteristics of adolescent mothers (*n* = 1640).

Variable	Category	n	%
Residence	Rural	1060	64.6
	Urban	580	35.4
Educational attainment	Primary (1–4 years)	702	42.8
	Lower secondary (5–8 years)	384	23.4
	Upper secondary (9–12 years/ongoing)	554	33.8
Mean maternal age (years)	—	16.3 ± 1.4	—

**Table 2:** Annual incidence of adolescent pregnancy (2014–2023).

Year	Total Births	Teenage Pregnancies	Incidence (%)
2014	1301	154	11.8
2015	1381	174	12.5
2016	1563	164	10.5
2017	1214	139	11.4
2018	1135	140	12.3
2019	1503	193	12.8
2020	1477	185	12.5
2021	1379	163	11.8
2022	1341	164	12.2
2023	1463	184	12.6
Total	13,416	1640	12.2

**Table 3:** Mode of delivery according to demographic characteristics.

Variable	Cesarean (%)	Vaginal (%)	*p* Value *
Overall (*n* = 1640)	58.3	41.7	—
Residence			
Rural	61.2	38.8	0.02
Urban	52.4	47.6	—
Education			
Primary	62.4	37.6	0.01
Secondary	57.7	42.3	—
Upper secondary	54.4	45.6	

* Significant at *p* < 0.05.

**Table 4:** Indications for cesarean delivery among adolescent mothers (*n* = 956).

Indication	n	%
Cervical dystocia (>37 weeks)	184	19.2
Cephalopelvic disproportion	162	16.9
Breech presentation	74	7.7
Fetal distress	70	7.3
Maternal comorbidities	38	4.0
Preterm complications (<37 weeks)	130	13.6
Previous uterine scar (≥1)	242	25.3
Other indications	56	5.9
Total	956	100.0

**Table 5:** Perinatal outcomes among adolescent pregnancies (*n* = 1640).

Outcome	n	%	Major Associated Factors	*p* Value *
Preterm birth (<37 weeks)	500	30.5	Rural residence; low education	0.016
Low birth weight (<2500 g)	444	27.1	Preterm delivery	<0.001
Apgar < 7 at 5 min	146	8.9	Preterm; cesarean	<0.001
NICU admission	190	11.6	Fetal distress; prematurity	<0.001

* Significant at *p* < 0.05.

**Table 6:** Multivariate variables associated with higher odds of cesarean delivery among adolescent pregnancies.

Variable	OR	95% CI	*p* Value *
Primiparity	2.10	1.45–3.05	<0.001
Low education	1.56	1.09–2.21	0.015
Rural residence	1.21	0.83–1.75	0.31
Gestational age (<37 weeks)	1.08	0.72–1.61	0.68

* Significant at *p* < 0.05.

## Data Availability

The data that support the findings of this study are available from the corresponding author upon reasonable request.

## Abbreviations

The following abbreviations are used in this manuscript:

WHO	World Health Organization
ICD	International Classification of Diseases
CSR	Cesarean Section Rate
STROBE	Strengthening the Reporting of Observational Studies in Epidemiology
PROM	Premature rupture of membranes
